# Clinical data on treatment regimen and use of medication among patients with hemophilia B in Korea

**DOI:** 10.1007/s44313-024-00024-8

**Published:** 2024-08-08

**Authors:** Young Shil Park, Ji Kyoung Park, Jeong A Park, Hee Jo Baek, Jae Hee Lee, Chur Woo You, Chuhl Joo Lyu, Eun Jin Choi

**Affiliations:** 1 Department of Pediatrics, Kyunghee University Hospital at Gandong, Seoul, Republic of Korea; 2 Department of Pediatrics, Busan Paik Hospital, Inje University College of Medicine, Busan, Republic of Korea; 3 Department of Pediatrics, Inha University College of Medicine, Incheon, Republic of Korea; 4 Department of Pediatrics, Chonnam National University Hwasun Hospital, Chonnam National University Medical School, Gwangju, Republic of Korea; 5 Chungbuk National University Hospital, Cheongju, Republic of Korea; 6 Daejeon Eulji Medical Center, Eulji University, Daejeon, Republic of Korea; 7 Severance Hospital, Yonsei University Health System, Seoul, Republic of Korea; 8 Department of Pediatrics, Daegu Catholic University Medical Center, Daegu, Republic of Korea

**Keywords:** Hemophilia B, Clinical data, Prophylaxis, Treatment regimen

## Abstract

**Background:**

To investigate the clinical treatment status, such as treatment regimen, bleeding events, and drug dose, in patients with hemophilia B in South Korea.

**Methods:**

In this retrospective chart review, data of patients with hemophilia B from eight university hospitals were collected. Demographic and clinical data, treatment data, such as regimen and number of injections, dose of factor IX concentrate, and bleeding data were reviewed. Descriptive analyses were performed with annual data for 2019, 2020, and 2021, as well as the three years consecutively.

**Results:**

The medical records of 150 patients with hemophilia B between January 1, 2019, and December 31, 2021, were collected. Among these, 72 (48.0%) were severe, 47 (31.3%) were moderate, and 28 (18.7%) were mild. The results showed approximately two times more patients receiving prophylaxis as those receiving on-demand therapy, with 66.1% of patients receiving prophylaxis in 2019, 64.9% in 2020, and 72.1% in 2021. Annualized bleeding rates were 2.2% (± 3.1) in 2019, 1.8% (± 3.0) in 2020, and 1.8% (± 2.9) in 2021 among patients receiving prophylaxis. For the doses of factor IX concentrate, patients receiving prophylaxis received an average of 41.6 (± 11.9) IU/Kg/Injection in 2019, 45.7 (± 12.9) IU/Kg/Injection in 2020, and 60.1 (± 24.0) IU/Kg/Injection in 2021.

**Conclusions:**

Clinically, prophylaxis is more prevalent than reported. Based on insights gained from current clinical evidence, it is expected that the unmet medical needs of patients can be identified, and physicians can evaluate the status of patients and actively manage hemophilia B using more effective treatment strategies.

**Supplementary Information:**

The online version contains supplementary material available at 10.1007/s44313-024-00024-8.

## Introduction

Hemophilia B is a congenital bleeding disorder characterized by a deficiency in blood coagulation factor IX [1]. The estimated prevalence was 3.8 cases per 100,000 males for all severities of hemophilia B based on national patient registry data from Australia, Canada, France, Italy, New Zealand, and the United Kingdom [2]. According to the Report on the Annual Global Survey 2021 of the World Federation of Hemophilia (WFH), the number of patients identified with hemophilia B was 37,998 worldwide, of whom 446 were from Korea [3].

Patients with hemophilia B can be treated with coagulation factor concentrates, depending on the treatment purpose: on-demand therapy for acute bleeding, prophylaxis for prevention, and immune tolerance induction therapy for coagulation factor inhibitors [4].

Coagulation factor replacement therapy has been possible since the 1960s, and with the introduction of plasma-derived coagulation factor concentrates, the management of hemophilia has improved radically since the 1970s [5].

In addition, the current treatment regimen focuses on prophylaxis rather than on-demand therapy [6]. There is strong evidence from randomized controlled trials and observational studies that prophylaxis preserves joint function in children with hemophilia compared with on-demand treatment [2]. The WFH strongly recommends that patients with severe hemophilia B receive prophylaxis [1].

A previous study reported that the proportion of patients receiving prophylaxis was high in patients with hemophilia in many developed countries. In the retrospective observational study, 92% of 25 patients with severe hemophilia B and 37.5% of 8 patients with moderate hemophilia B received prophylaxis from 2017 to 2021, in Germany [7]. With advances in treatment of hemophilia, evidence is being generated in clinical practice of patients with hemophilia worldwide; however, there is a lack of studies on patients with hemophilia B in Korea.

Although hemophilia B is rare, it is associated with a significant economic burden. According to a report by the Health Insurance Review and Assessment Service in Korea, the average annual claim per patient for all prescribed blood coagulation factors is approximately 100 million won, which has a significant impact on health insurance costs [8]. However, there have been no studies on the practice treatment patterns of patients with hemophilia B in a clinical setting in Korea. Therefore, we aimed to investigate clinical data of patients with hemophilia B in Korea. The primary objective of this study was to identify the percentage of patients with hemophilia B receiving prophylaxis. The secondary objective was to understand the status of healthcare resource utilization and bleeding-related information among Korean patients with hemophilia B.

## Material and methods

### Study design

This multicenter retrospective chart review was conducted on data from eight university hospitals in Korea. Patient demographics and clinical data, including sex, age, severity of the disease, were collected to align with the study objectives. In addition, treatment status information (prophylaxis, on-demand therapy) and use of medical services information (number of outpatient visits or hospitalizations, and annual drug prescription dose) were collected.

Institutional Review Board approval was obtained at each hospital for conducting this study.

### Study subjects

Patients who had medical records showing hemophilia B at least once between January 1, 2019, and December 31, 2021, at the eight sites were eligible. Patients without available medical records were excluded. Patients with an historical antibody value of 0.6 BU or higher and confirmed positive for antibodies were also excluded. Patients who visited the outpatient clinic for counseling, surgery, and bleeding management were also included, even if they were not prescribed medication to treat hemophilia at the medical centers.

Patients were classified into severe, moderate, and mild according to the activity of the blood coagulation factor identified at the time of diagnosis of hemophilia B. Severity of hemophilia B was defined as follows: Severe: coagulation factor activity < 1% or < 1 IU/dL (< 0.01 IU/mL); Moderate: coagulation factor activity of 1–5% or 1–5 IU/dL (0.01–0.05 IU/mL); Mild: coagulation factor activity > 5% or 5–40 IU/dL (0.05–0.40 IU/mL). Regarding the treatment regimen, the investigator assessed each patient as either receiving prophylaxis or receiving on-demand therapy, and the classification was based on the patient's treatment pattern in a clinical setting. The drug dose was calculated per unit weight and the number of injections (IU/Kg/Injection) of the prescribed formulation for each year. Descriptive analyses were performed with annual data for 2019, 2020, and 2021, as well as the three years consecutively.

## Methods

For annualized bleeding rate (ABR) analysis, patients who were either 1) prescribed primarily by another hospital or 2) documented as switching from on-demand treatment to prophylaxis or prophylaxis to on-demand treatment in the following year were assessed for exclusion.

To ensure constant prophylaxis for three years, an operational definition was applied: prophylaxis for three consecutive years and prescribed a drug dose of at least 40 IU/Kg/Injection and above per year. Patients were not included in the consecutive prophylaxis group if the type of agent administration was changed; for example, if a standard half-life (SHL) agent was prescribed in 1st half of the year and then an extended half-life (EHL) agent was prescribed in 2nd half of the year.

### Analysis

The demographic and clinical data collected through a retrospective review of the medical records of each hospital were summarized using descriptive statistics. Continuous variables are presented as mean ± standard deviation and categorical variables as frequency (percentage). No other imputation method was used for missing values. All analyses were performed using SAS Software version 9.4.

## Results

### Patient’s characteristics

A total of 150 patients with hemophilia B with medical records between January 1, 2019, and December 31, 2021, were collected from eight university hospitals participating in the study. Of the 150 patients, 147 were male and three were female, with a mean age of 28.8 ± 19.3 years. By age group, patients aged 20 to 40 years comprised the largest group (31.3%). Comorbidities included osteonecrosis in nine patients, chronic hepatitis C in seven, life-threatening bleeding with intracranial hemorrhage in four, and intra-abdominal bleeding in four. There were 72 (48.0%) patients with severe hemophilia B, 47 (31.3%) had moderate hemophilia B, and 28 (18.7%) had mild hemophilia B. Three patients had no information on the severity of hemophilia B recorded in their medical records (Table 1).

**Table 1:** Baseline demographic and clinical characteristics of study participants, 2019–2021 (*N* = 150)

Variables	All patients	
	**n**	**%**
**Sex**		
**Female**	3	2.0
**Male**	147	98.0
**Age (years)**^**a**^		
**Mean (SD)**	28.8	(19.3)
**Median**	23	
**Min**	1	
**Max**	86	
**Age Group**		
** < 12 years**	33	22.0
** > = 12 to < 20 years**	29	19.3
** > = 20 to < 40 years**	47	31.3
** > = 40 to < 60 years**	29	19.3
** > = 60 years**	12	8.0
**Blood Types (Category)**		
**O**	25	17.0
**Non-O (A, B, and AB)**	72	48.0
**UK**	53	35.0
**Weight (Kg, at the most recent visit)**		
**N**	146	
**Mean (SD)**	62.3	(24.8)
**Median**	64.3	
**Min**	10.0	
**Max**	120.0	
**Medical status (allows duplication)**		
**Acquired immunodeficiency syndrome**	0	
**Chronic bronchitis**	2	
**Chronic hepatitis B**	2	
**Chronic hepatitis C / Hepatitis C**	7	
**Hepatitis C RNA negative (-)**	5	
**Not reported RNA results**	2	
**Diabetes mellitus**	0	
**Hemophilic arthropathy**	3	
**Hypertension**	0	
**Osteonecrosis**	9	
**Experience of life-threatening bleeding (baseline history)**		
**Intracranial Hemorrhage**	4	2.7
**Intra-abdominal Hemorrhage**	4	2.7
**Severity of Hemophilia B (*N* = 150)**		
Mild	28	18.7
Moderate	47	31.3
Severe	72	48.0
NR	3	2.0

*NR* not reported, *UK* Unknown

^a^Age was calculated as of 01 September, 2022

### Treatment regimen

In 2019, 62 patients were prescribed coagulation factor agents as prophylaxis or on-demand therapy at each hospital with 74 in 2020, and 68 in 2021. There were approximately two times more patients who received prophylaxis than those who received on-demand treatment, with 41 patients (66.1%) receiving prophylaxis in 2019, 48 (64.9%) in 2020, and 49 (72.1%) in 2021.

### Annualized bleeding rates (ABR) and drug dose among patients receiving prophylaxis

For patients who received prophylaxis the mean ABRs for 2019, 2020 and 2021 were 2.2 ± 3.1 (*n* = 38), 1.8 ± 3.0 (*n* = 45), and 1.8 ± 2.9 (*n* = 45), respectively (Table 2). For patients with severe hemophilia B who received prophylaxis, the mean ABRs for 2019, 2020, and 2021 were 2.6 ± 3.5 (*n* = 27), 2.4 ± 3.4 (*n* = 30), and 1.7 ± 2.8 (*n* = 31), respectively. In addition, patients who received prophylaxis had minimum ABRs of 0 in all three years and maximum ABRs of 12, 14, and 13 in 2019, 2020, and 2021, respectively (Table 2).

**Table 2:** Annualized bleeding rates (ABR) and drug dose among patients receiving prophylaxis

		**Mean**	**(SD)**	**Median**	**Min**	**Max**
**ABR**						
**2019 (*n* = 38)**	**Total**	**2.2**	**3.1**	**1.0**	**0**	**12.0**
	Mild (*n* = 1)	4.0	-	-	-	-
	Moderate (*n* = 10)	0.9	1.0	0.5	0	3.0
	Severe (*n* = 27)	2.6	3.5	1.0	0	12.0
**2020 (*n* = 45)**	**Total**	**1.8**	**3.0**	**1.0**	**0**	**14.0**
	Mild (*n* = 3)	1.3	1	1.0	0	3.0
	Moderate (*n* = 12)	0.6	0.9	0	0	3.0
	Severe (*n* = 30)	2.4	3.4	1.0	0	14.0
**2021 (*n* = 45)**	**Total**	**1.8**	**2.9**	**1.0**	**0**	**13.0**
	Mild (*n* = 2)	3.5	3.5	3.5	0	7.0
	Moderate (*n* = 12)	1.5	3.0	0.5	0	11.0
	Severe (*n* = 31)	1.7	2.8	1.0	0	13.0
**Dose**^a^** (IU/Kg/1 injection)**						
2019	Total (*n* = 38)	41.6	11.9	41.5	22.4	85.9
2020	Total (*n* = 39)	45.7	12.9	43.1	18.4	79.8
2021	Total (*n* = 37)	60.1	24.0	52.1	32.2	128.9

^a^Only patients who were receiving the standard half-life (SHL) agents in each year was analyzed

Among the patients who received prophylaxis, some switched to an EHL agent. Thus, only patients who received an SHL agent in each year were analyzed for drug dose calculation. For the dose analysis there were 38 patients in 2019, 39 in 2020, and 37 in 2021 and they received mean doses of 41.6 ± 11.9 IU/Kg/Injection, 45.7 ± 12.9 IU/Kg/Injection, and 60.1 ± 24.0 IU/Kg/Injection, respectively (Table 2).

### Annualized bleeding rates (ABR) and drug dose patients who received on-demand therapy

Among patients with hemophilia B who received on-demand treatment, the mean ABR was 2.2 ± 3.2 in 2019, 1.9 ± 3.0 in 2020, and 1.8 ± 1.8 in 2021. The mean injection dose of SHL agents was 41.0 ± 16.1 IU/Kg/Injection in 2019, 55.6 ± 43.6 IU/Kg/Injection in 2020, and 55.3 ± 23.5 IU/Kg/Injection in 2021 (Table 3).

**Table 3:** Annualized bleeding rates (ABR) and drug dose among patients receiving on-demand treatment

		**Mean**	**(SD)**	**Median**	**Min**	**Max**
**ABR**						
**2019 (*n* = 19)**	**Total**	**2.2**	**3.2**	**1.0**	**0**	**14.0**
	Mild (*n* = 5)	0.8	0.4	1.0	0	1
	Moderate (*n* = 7)	2.6	4.7	1.0	0	14.0
	Severe (*n* = 7)	2.8	2.0	2.0	0	7.0
**2020 (*n* = 26)**	**Total**	**1.9**	**3.0**	**1.0**	**0**	**14.0**
	Mild (*n* = 10)	0.9	0.7	1.0	0	2.0
	Moderate (*n* = 9)	2.3	4.2	1.0	0	14.0
	Severe (*n* = 7)	2.9	3.0	2.0	0	10.0
**2021 (*n* = 19)**	**Total**	**1.8**	**1.8**	**1.0**	**0**	**8.0**
	Mild (*n* = 4)	0.8	0.4	1.0	0	1.0
	Moderate (*n* = 7)	2.3	2.5	1.0	0	8.0
	Severe (*n* = 8)	2.0	1.1	1.5	0	4
**Dose**^a^** (IU/Kg/1 injection)**						
2019	Total (*n* = 19)	41.0	16.1	37.7	15.9	77.8
2020	Total (*n* = 26)	55.6	43.6	46.8	23.4	228.2
2021	Total (*n* = 19)	55.3	23.5	47.7	31.0	134.2

^a^Only patients who were receiving the standard half-life (SHL) agents in each year was analyzed

### Patients with hemophilia B who received prophylaxis for three consecutive years (2019–2021)

Another subgroup was identified as having received prophylaxis consistently for three consecutive years (2019–2021). Thus, 24 patients who received prophylaxis for three consecutive years were included in this analysis. The baseline characteristics of the 24 patients are shown in Supplementary Table 2. All patients were male, with a mean age of 21.6 ± 18.5 years. When stratified by severity of hemophilia B, six (24.0%) had moderate disease and 18 (76.0%) had severe disease. The dose per weight per injection over the course of treatment in the 24 patients who received prophylaxis for three consecutive years is presented in Table 4. The mean ABR of the three years was 2.0 ± 3.1. Specifically in 2021, the mean drug dose was higher (62.9 IU/Kg/Injection) compared to the other years (41.8 IU/Kg/Injection in 2019, 44.5 IU/Kg/Injection in 2020) and there was a lower mean ABR (1.5) compared to the other years (2.3 in 2019 and 2020, not statistically significant).

**Table 4:** Dose among patients receiving prophylaxis during three consecutive years (*n* = 24)

	**Mean**	**(SD)**	**Median**	**Min**	**Max**
**ABR**					
3-year average^a^	2.0	(3.1)	0.7	0	13
2019	2.3	(3.5)	1.0	0	12
2020	2.3	(3.3)	1.0	0	14
2021	1.5	(3.0)	0.0	0	13
**Dose (IU/Kg/1 injection)**					
2019	41.8	(9.3)	41.5	23.0	64.2
2020	44.5	(8.5)	44.8	26.0	57.7
2021	62.9	(28.2)	51.5	32.2	128.9

^a^Average of 3-year average ABR per patient

## Discussion

This study retrospectively collected medical records of 150 patients with hemophilia B from eight university hospitals in Korea to investigate their treatment status. The primary objective of this study was to determine the percentage of patients who received prophylaxis each year from 2019 to 2021. A higher percentage of patients received prophylaxis than on-demand therapy, with 41 of 62 patients (66.1%) receiving confirmed prophylaxis in 2019, 48 of 74 (64.9%) in 2020, and 49 of 78 (72.1%) in 2021. 

In a retrospective observational study conducted in the United States, 72.9% (675 of 926) of patients with severe hemophilia B received prophylaxis in 2018, compared with 55% in 2015, an increase of approximately 33% [9]. Furthermore, of the 300 patients receiving prophylaxis with EHL coagulation factors, 63.3% reported received their medication every week, 12.7% every 10 days, and 15.0% every two weeks [9].

Another study conducted in three European countries also found a high percentage of patients receiving prophylaxis. In the study of patients with hemophilia B using EHL recombinant FIX (rFIX) products in Italy, Belgium, and the United Kingdom, the percentage of patients receiving prophylaxis prior to using EHL products ranged from 70 to 89.8%, and 100% of patients received prophylaxis after using EHL products [10].

In contrast to earlier findings, the annual report released by the Korean Hemophilia Foundation indicates that the use of prophylaxis in patients with hemophilia B was 40.3% (175 of 434) in 2019 44.7% (196 of 438) in 2020, and 35.7% (159 of 446) in 2021 [11–13]. These data are lower than those observed in clinical data, implying that a larger percentage of patients in clinical practice receive prophylaxis.

Among the findings of this study, there was no significant difference in ABR between patients who received prophylaxis and those treated with on-demand therapy, in contrast to previous studies that reported lower ABR in patients who received prophylaxis [14, 15]. This may be due to limitations in obtaining information arising from the study design that reviewed existing medical records. For example, there was no approach for identifying bleeding that was not documented in the medical charts; therefore, there was limited information related to bleeding in the records. As the study only identified healthcare use, such as number of injections and drug dose, at the participating hospital, it was limited in identifying information in other healthcare institutions for bleeding management or medication. This suggests that the ABR in this study may not have been accurately estimated. However, the results of the current study for the ABR were similar to those of other studies. Ay et al. [14], reported a median ABR (joint bleeding) of 2.0 (IQR 0.8–6.4) in patients with severe hemophilia B receiving prophylaxis. In addition, Berntorp et al. [15] reported the ABRs in patients with severe hemophilia B in European countries, with a median ABR (all sites) ranging from 0 (Germany) to 6.0 (Belgium).

This study did not find a significant difference in ABRs between patients who received prophylaxis and those treated with on-demand therapy. There are two possible clinical explanations for this finding. First, the dose administered during prophylaxis may have been insufficient to prevent patients with hemophilia B from bleeding. Second, that patients may have underreported bleeding because of differences in treatment methods across hospitals, how physicians reported treatment information, or how patients reported their bleeding experiences.

Among 24 patients classified as receiving consecutive prophylaxis, as per the operational criteria, the dosages administered per injection were 41.8 IU/Kg in 2019, 44.5 IU/Kg in 2020, and 62.9 IU/Kg in 2021. The relatively high mean dosage in 2021 can be attributed to variation in prophylaxis regimens, with some centers opting for a dosage range of 40–50 IU/kg/Injection twice weekly, whereas others adopted a dosage range of 80–100 IU/kg/Injection once weekly.

This study is significant because it reviewed the treatment status of patients with hemophilia B in Korea, including treatment regimens, number of bleeds, number of injections, and doses in clinical practice. Previous studies have emphasized the importance of examining the clinical impact of new clotting factor agents as treatment methods for hemophilia continue to evolve [9]; however, to date, no study has reported the clinical treatment of patients with hemophilia B in Korea. This study represents clinical treatment of patients with hemophilia B in Korea because it included data from the medical records of general hospitals specializing in the treatment of hemophilia. It has been reported that hemophilia B is less prevalent than hemophilia A, and many hemophilia studies have focused on only on patients with hemophilia A, or on the clinical burden and outcomes of the entire hemophilia A and B cohort (comprised mostly of patients with hemophilia A). However, the clinical and economic burdens of hemophilia B are substantial, persistent, and require attention [16].

In conclusion, there is an ongoing requirement for long-term observational studies. In the clinic, prophylaxis is more prevalent than has been previously reported. For optimized prophylaxis, personalization should be further studied by analyzing the bleeding phenotype and lifestyle of patients. Based on insights gained from current clinical evidence, it is expected that the unmet medical needs of patients can be identified and physicians can evaluate the status of patients and actively manage hemophilia B using more effective treatment strategies.

### Supplementary Information

Additional file 1: Supplementary Table 1. IRB approval number of each participating hospital. Supplementary Table 2. Baseline characteristics of the patients receiving prophylaxis for three consecutive years (*n*=24).

## Data Availability

The data that support the findings of this study are available from CSL Behring, but restrictions apply to the availability of these data, which were used under license for the current study and so are not publicly available. The data are, however, available from the authors upon reasonable request and with the permission of CSL Behring.
