# Supplementary material for: Clinical data on treatment regimen and use of medication among patients with hemophilia B in Korea
Source: Blood Res. 2024 Aug 8;59(1):27. doi: 10.1007/s44313-024-00024-8 (PMC11310372; doi:10.1007/s44313-024-00024-8)
Supplement: Supplementary file 1 — Additional file 1: Supplementary Table 1. IRB approval number of each participating hospital. Supplementary Table 2. Baseline characteristics of the patients receiving prophylaxis for three consecutive years (n=24). [file 44313_2024_24_MOESM1_ESM.docx]

Table 1. Baseline demographic and clinical characteristics of study participants, 2019-2021 (N=150)

| **Variables** | **All patients** | |
| --- | --- | --- |
|  | **n** | **%** |
| **Sex** |  |  |
| Female | 3 | 2.0 |
| Male | 147 | 98.0 |
| **Age (years)*** |  |  |
| Mean (SD) | 28.8 | (19.3) |
| Median | 23 |  |
| Min | 1 |  |
| Max | 86 |  |
| **Age Group** |  |  |
| <12 years | 33 | 22.0 |
| >=12 to <20 years | 29 | 19.3 |
| >=20 to <40 years | 47 | 31.3 |
| >=40 to <60 years | 29 | 19.3 |
| >=60 years | 12 | 8.0 |
| **Blood Types (Category)** |  |  |
| O | 25 | 17.0 |
| Non-O (A, B, and AB) | 72 | 48.0 |
| UK | 53 | 35.0 |
| **Weight (Kg, at the most recent visit)** |  |  |
| N | 146 |  |
| Mean (SD) | 62.3 | (24.8) |
| Median | 64.3 |  |
| Min | 10.0 |  |
| Max | 120.0 |  |
| **Medical status (allows duplication)** |  |  |
| Acquired immunodeficiency syndrome | 0 |  |
| Chronic bronchitis | 2 |  |
| Chronic hepatitis B | 2 |  |
| Chronic hepatitis C / Hepatitis C | 7 |  |
| Hepatitis C RNA negative (-) | 5 |  |
| Not reported RNA results | 2 |  |
| Diabetes mellitus | 0 |  |
| Hemophilic arthropathy | 3 |  |
| Hypertension | 0 |  |
| Osteonecrosis | 9 |  |
| **Experience of life-threatening bleeding (baseline history)** | | |
| Intracranial Hemorrhage | 4 | 2.7 |
| Intra-abdominal Hemorrhage | 4 | 2.7 |
| **Severity of Hemophilia B(N=150)** |  |  |
| Mild | 28 | 18.7 |
| Moderate | 47 | 31.3 |
| Severe | 72 | 48.0 |
| NR | 3 | 2.0 |
| NR not reported, UK Unknown | | |

*Age was calculated as of 01 September, 2022.

Table 2. Annualized bleeding rates (ABR) and drug dose among prophylaxis patients

|  |  | **Mean** | **(SD)** | **Median** | **Min** | **Max** |
| --- | --- | --- | --- | --- | --- | --- |
| **ABR** |  |  |  |  |  |  |
| **2019 (n=38)** | **Total** | **2.2** | **3.1** | **1.0** | **0** | **12.0** |
|  | Mild (n=1) | 4.0 | - | - | - | - |
|  | Moderate (n=10) | 0.9 | 1.0 | 0.5 | 0 | 3.0 |
|  | Severe (n=27) | 2.6 | 3.5 | 1.0 | 0 | 12.0 |
| **2020 (n=45)** | **Total** | **1.8** | **3.0** | **1.0** | **0** | **14.0** |
|  | Mild (n=3) | 1.3 | 1. | 1.0 | 0 | 3.0 |
|  | Moderate (n=12) | 0.6 | 0.9 | 0 | 0 | 3.0 |
|  | Severe (n=30) | 2.4 | 3.4 | 1.0 | 0 | 14.0 |
| **2021 (n=45)** | **Total** | **1.8** | **2.9** | **1.0** | **0** | **13.0** |
|  | Mild (n=2) | 3.5 | 3.5 | 3.5 | 0 | 7.0 |
|  | Moderate (n=12) | 1.5 | 3.0 | 0.5 | 0 | 11.0 |
|  | Severe (n=31) | 1.7 | 2.8 | 1.0 | 0 | 13.0 |
| **Dose* (IU/Kg/1 injection)** | | | | | | |
| 2019 | Total (n=38) | 41.6 | 11.9 | 41.5 | 22.4 | 85.9 |
| 2020 | Total (n=39) | 45.7 | 12.9 | 43.1 | 18.4 | 79.8 |
| 2021 | Total (n=37) | 60.1 | 24.0 | 52.1 | 32.2 | 128.9 |

* Only patients who were receiving the standard half-life (SHL) agents in each year was analyzed.

Table 3. Annualized bleeding rates (ABR) and drug dose among on-demand patients

|  |  | **Mean** | **(SD)** | **Median** | **Min** | **Max** |
| --- | --- | --- | --- | --- | --- | --- |
| **ABR** |  |  |  |  |  |  |
| **2019 (n=19)** | **Total** | **2.2** | **3.2** | **1.0** | **0** | **14.0** |
|  | Mild (n=5) | 0.8 | 0.4 | 1.0 | 0 | 1 |
|  | Moderate (n=7) | 2.6 | 4.7 | 1.0 | 0 | 14.0 |
|  | Severe (n=7) | 2.8 | 2.0 | 2.0 | 0 | 7.0 |
| **2020 (n=26)** | **Total** | **1.9** | **3.0** | **1.0** | **0** | **14.0** |
|  | Mild (n=10) | 0.9 | 0.7 | 1.0 | 0 | 2.0 |
|  | Moderate (n=9) | 2.3 | 4.2 | 1.0 | 0 | 14.0 |
|  | Severe (n=7) | 2.9 | 3.0 | 2.0 | 0 | 10.0 |
| **2021 (n=19)** | **Total** | **1.8** | **1.8** | **1.0** | **0** | **8.0** |
|  | Mild (n=4) | 0.8 | 0.4 | 1.0 | 0 | 1.0 |
|  | Moderate (n=7) | 2.3 | 2.5 | 1.0 | 0 | 8.0 |
|  | Severe (n=8) | 2.0 | 1.1 | 1.5 | 0 | 4 |
| **Dose* (****IU/Kg/1 injection)** | | | | | | |
| 2019 | Total (n=19) | 41.0 | 16.1 | 37.7 | 15.9 | 77.8 |
| 2020 | Total (n=26) | 55.6 | 43.6 | 46.8 | 23.4 | 228.2 |
| 2021 | Total (n=19) | 55.3 | 23.5 | 47.7 | 31.0 | 134.2 |

* Only patients who were receiving the standard half-life (SHL) agents in each year was analyzed.

Table 4. Annualized bleeding rates (ABR) and dose among prophylaxis patients during 3 consecutive years (n=24)

|  | **Mean** | **(SD)** | **Median** | **Min** | **Max** |
| --- | --- | --- | --- | --- | --- |
| **ABR** |  |  |  |  |  |
| 3-year average* | 2.0 | (3.1) | 0.7 | 0 | 13 |
| 2019 | 2.3 | (3.5) | 1.0 | 0 | 12 |
| 2020 | 2.3 | (3.3) | 1.0 | 0 | 14 |
| 2021 | 1.5 | (3.0) | 0.0 | 0 | 13 |
| **Dose (IU/Kg/1 injection)** | | | | | |
| 2019 | 41.8 | (9.3) | 41.5 | 23.0 | 64.2 |
| 2020 | 44.5 | (8.5) | 44.8 | 26.0 | 57.7 |
| 2021 | 62.9 | (28.2) | 51.5 | 32.2 | 128.9 |
| *Average of 3-year average ABR per patient | | | | | |

**Supplementary Table 1. IRB approval number of each participating hospital**

| **Hospital name** | **IRB approval number** |
| --- | --- |
| Daegu Catholic University Medical Center | CR-22-119 |
| Chonnam National University Hwasun Hospital | CNUHH-2022-162 |
| Inje University Busan-Paik Hospital | BPIRB 2022-08-051 |
| Kyung Hee University Hospital at Gangdong | 2022-09-015 |
| Severance Hospital, Yonsei University Health System | 4-2022-1107 |
| Daejeon Eulji Medical Center | 2022-11-009 |
| Chungbuk National University Hospital | 2022-10-025 |
| Inha University Hospital | 2022-10-024 |

Supplementary Table 2. Baseline characteristics of the prophylaxis patients for 3 consecutive years (n=24)

| **Variables** | **All patients** | |
| --- | --- | --- |
|  | **n** | **%** |
| **Sex** |  |  |
| Male | 24 | 100.0 |
| **Age (year)*** |  |  |
| Mean (SD) | 21.6 | (18.5) |
| Median | 13 |  |
| **Age Group** |  |  |
| <12 years | 10 | 41.7 |
| >=12 to <20 years | 6 | 25.0 |
| >=20 to <40 years | 4 | 16.7 |
| >=40 to <60 years | 2 | 8.3 |
| >=60 years | 2 | 8.3 |
| **Severity Of Hemophilia B** |  |  |
| Mild | 0 | 0.0 |
| Moderate | 6 | 24.0 |
| Severe | 18 | 76.0 |
| **Blood Types (Category)** |  |  |
| O | 4 | 16.7 |
| Non-O (A, B, AB) | 12 | 50.0 |
| UK | 8 | 33.3 |
| **Medical status (allows duplication)** |  |  |
| Acquired immunodeficiency syndrome | 0 |  |
| Chronic hepatitis C / Hepatitis C | 2 |  |
| Hepatitis C RNA negative (-) | 2 |  |
| Not reported RNA results | 0 |  |
| Chronic hepatitis B | 0 |  |
| Hypertension | 2 |  |
| Hemophilic arthropathy | 2 |  |
| Osteonecrosis | 0 |  |
| Diabetes mellitus | 1 |  |
| Chronic bronchitis | 0 |  |
| **Experience of life threatening bleeding (baseline history)** | | |
| Intracranial Hemorrhage | 1 | 4.1 |
| Intra-abdominal Hemorrhage | 0 |  |
| UK Unknown | | |

*Age was calculated as of 01 September, 2022.
